# Factors affecting the quality of life in pregnant women with diabetes: the mediating effect of illnesses acceptance

**DOI:** 10.1186/s12884-024-06690-x

**Published:** 2024-07-29

**Authors:** Azita Fathnezhad-Kazemi, Zahra Seifinadergoli, Mohaddeseh Ahmadi

**Affiliations:** 1 Department of Midwifery, Women’s Reproductive and Mental Health Research Center, Tabriz Medical Sciences, Islamic Azad University, Tabriz, Iran; 2 Department of Midwifery, Faculty of Medical Sciences, Tabriz Medical Sciences, Islamic Azad University, Tabriz, Iran; 3 Students’ Research Committee, Faculty of Medicine, Tabriz Medical Sciences, Islamic Azad University, Tabriz, Iran

**Keywords:** Illnesses acceptance, Quality of life, Pregnancy, Self-efficacy, Hyperglycemia

## Abstract

**Introduction:**

Gestational diabetes, as a disorder of carbohydrate metabolism, is considered one of the most common metabolic complications in pregnancy. The diagnosis of diabetes in pregnancy leads to changes in lifestyle, and the treatments employed can affect various aspects of pregnant women’s lives, including their quality of life. The present study aimed to investigate the relationship between self-efficacy and the mediating effect of illness acceptance on the quality of life in pregnant women diagnosed with diabetes during pregnancy.

**Materials and methods:**

This cross-sectional study was conducted on 240 pregnant women diagnosed with diabetes who were selected by convenience sampling method. Quality of life tools (SF12), the Acceptance of Illness Scale (AIS), and the Sherer self-efficacy scale were used to collect data.

**Results:**

The mean (SD) of quality of life, self-efficacy, and disease acceptance were 57.36 (6.63), 51.75 (7.44), and 29.07 (7.69), respectively. In the single-variable regression analysis, self-efficacy and disease acceptance variables could predict 20.6% (β = 0.457, *P* < 0.001) and 14.4% (β = 0.385, *P* < 0.001) of the variations in quality of life, respectively. In the multiple regression model, by entering the two main variables (self-efficacy and Acceptance of Illness), demographic characteristics, three disease knowledge variables, health status from an individual perspective, and type of treatment, the variables could explain 25% of the changes of the quality of life (R^2^_adj_ 0.25, *P* < 0.001=). Income status and self-efficacy had the highest impact among the variables. According to the results of path analysis, the total effect of self-efficacy on the quality of life of pregnant women with diabetes was 0.711.

**Conclusion:**

The overall quality of life in women with diabetes was moderate, and self-efficacy, illness acceptance, and income status were predictors of overall quality of life. Self-efficacy can influence the quality of life by affecting disease acceptance. The findings highlight the importance of designing educational programs and providing midwifery services to increase self-efficacy and illness acceptance to improve the quality of life of pregnant women with diabetes.

## Introduction

Diabetes is one of the most common metabolic complications of pregnancy [1]. Research on the epidemiology of diabetes indicates a steady rise in the number of women affected by carbohydrate metabolism disorders during pregnancy [2]. According to information reported in 2017 by the International Diabetes Federation (IDF), 16.2% of pregnancies are complicated by hyperglycemia, with gestational diabetes mellitus (GDM) accounting for 86.4% of cases [3, 4]. Other sources have reported that hyperglycemia occurs as a pregnancy complication in 1–30% of cases worldwide [5]. This wide variability in the prevalence of hyperglycemia in pregnant women is due to heterogeneous protocols for diagnosing and classifying hyperglycemic disorders in different regions of the world [6, 7]. Regardless of the type of hyperglycemia occurring during pregnancy, the risk of complications increases for both the pregnant mother and her offspring, affecting their future health [8]. The occurrence of carbohydrate metabolism disorders during pregnancy requires lifestyle changes for the patient or, in some cases, pharmacological treatment, which may affect the perceived quality of life of the patient [3, 9]. Like other chronic diseases, diabetes can negatively impact almost all aspects of a patient’s life [10]. It often leads to worsening physical and mental health of the patient and brings about changes in lifestyle and adaptation to the disease, as well as changes in physical, professional, and social activities, which also affect the patient’s quality of life [7, 11]. Quality of life is determined by an individual’s perception of their life situation in terms of culture, values, goals, interests, expectations, and personal standards [12]. Quality of life encompasses four health-related dimensions: physical health, psychological status, social relationships, and the living environment [13]. Research related to quality of life has been widely used to gain a comprehensive understanding of individuals’ health status, yet existing studies have mainly been conducted in developed countries [14]. Therefore, examining the quality of life in developing countries, especially in high-risk populations such as pregnant women, is essential. In Iran, a study revealed that patients with type 2 diabetes had a moderate quality of life [15]. In another study, diabetic patients were found to have a particularly poor quality of life [16]. A study by Chinese researchers has shown that the HRQoL (Health-Related Quality of Life) among Chinese T2DM (Diabetes mellitus Type 2) patients may be impaired by decreased self-efficacy and poor glycemic control [17]. Various factors can influence quality of life, and demographic characteristics such as young maternal age, place of birth, and specific race (e.g., Asian or Black individuals) are associated with poor quality of life [11, 18]. Furthermore, evidence suggests that disease acceptance, stress, and coping strategies are important predictors of diseases and the physical and mental well-being of patients [19]. Disease acceptance is defined as the absence of negative responses and emotions associated with conditions. The stages of disease acceptance are a complex process [20]. Individuals faced with a diagnosis of a disease will experience grief and sorrow in the process of accepting the disease and may encounter multiple challenges, which can, in turn, lower their quality of life [21]. Moreover, in this disease, self-management and self-care for maintaining normal blood glucose levels and the health of the mother and fetus are considered very important, for example, researchers have reported that diabetic patients who can manage their health tend to have a better quality of life [22], in addition, capable people who have awareness and the ability to make decisions to improve their physical and mental health will have a better quality of life [23], and self-efficacy is one of the determinants of self-management in diseases [24, 25]. Self-efficacy is a belief in one’s ability to overcome challenges [25]. General self-efficacy is a belief that an individual can act specifically [26]. Evidence shows that a high level of general self-efficacy is a key factor in making lifestyle changes, leading to changes in the physical aspect of quality of life [3, 27]. Research has shown that self-efficacy is a significant predictor of disease acceptance and the psychological aspect of quality of life in patients with multiple sclerosis [28]. The patient’s adaptation to problems and changes in lifestyle associated with the disease, acceptance of the disease, and self-efficacy in patients may enhance the effectiveness of care for these patients and also increase their quality of life. Since research in this area is limited to pregnant mothers with diabetes, the present study was designed to examine the relationship between self-efficacy and the mediating effect of illness acceptance on the quality of life in pregnant women diagnosed with diabetes during pregnancy.

## Method

### Study design and setting

The present study is a descriptive-analytical cross-sectional study, which was conducted from April to late December 2023 in the obstetrics and gynecology clinics of two hospitals in Tabriz City, Iran. Both hospitals are important centers for providing antenatal care and are considered academic and educational centers.

### Study participants, sample size, and sampling

The inclusion criteria for the study included: 1- Women aged 18 years and older, 2- Singleton pregnant women with an initial diagnosis of diabetes during pregnancy who did not know a previous history of having diabetes, 3- Literacy (ability to read and write), 4- No use of antidepressant medications, 5- Absence of other chronic diseases such as hypertension, thyroid disease, and liver disease. The criteria for exiting the study included: failure to complete more than 10% of the questionnaire items and dissatisfaction with continuing the study. Hyperglycemia was diagnosed as follows: (A) Gestational diabetes: (fasting glucose level of 92–125 mg/dL and/or 180 mg/dL after 60 min and/or 153–199 mg/dL after 120 min), (B) Diabetes in pregnancy: Diabetes in pregnancy (DIP) is diagnosed when at least one of the following criteria is met: fasting glucose over 126 mg/dL, glucose level at 2 h in 75 g OGTT of 200 mg/dl, or random glucose level over 200 mg/dL with clinical symptoms of hyperglycemia [9].

The sample size was calculated based on the formula and the study’s results by Dalfra et al. [11] for the physical performance score of quality of life. Initially, 216 cases were calculated, and finally, with a 10% increase in study accuracy, the final sample size was considered as 240 cases.

Study parameter: alpha = 0.05, power = 0.90, m = 68.0, SD = 25.5, d = 5% mean

$$\:=\frac{{({Z}_{1}-\frac{\alpha\:}{2})}^{2}\:\times\:{s}^{2}}{{d}^{2}}$$

The participants were selected using the convenience sampling method. Sampling continued until the calculated sample size was reached. After obtaining the necessary ethical code and permission for sampling, the researcher attended the obstetrics and gynecology clinic and recruited eligible pregnant women based on the study criteria. After explaining the study objectives to pregnant women and obtaining their informed consent, the questionnaires for self-reporting were provided to them. If any issues or questions arose while completing the questionnaire, the researcher offered the necessary guidance.

### Data collection tools

1- **The demographic and obstetric questionnaire** included questions about age, level of education, employment status, income level, parity, gestational age, health status, treatment method, and individual knowledge about the disease which was obtained through their personal opinions and self-reporting, categorized as high, medium, and low awareness levels.

#### 2- Health-related quality of life (SF-12)

Ware, et al., designed the Short-Form Health Survey (SF-12) [29], it is a shortened form of the SF-36 Health Survey (SF-36) [30], is a widely used instrument for assessing patient-reported general health conditions/ Health-Related Quality of Life. The instrument is categorized into eight health domains to evaluate physical and mental health, each including six items. Physical health scales include general health (1 item), physical functioning (2 items), role physical (2 items), and bodily pain (1 item). Mental health domains include vitality (1 item), social functioning (1 item), role emotional (2 items), and mental health (2 items). Scores for items range from 1 to 6. To enable comparison of the study results in different cultures, we used US population-derived SF-12 norms which consider a mean value of 50 and a standard deviation value of 10 [31]. Scores on this questionnaire are in the range of 0–100, where higher scores indicate a better self-perceived health status. The validity and reliability of this questionnaire in Iran have been evaluated by Montazeri et al. [32].

**3- Illness Acceptance Questionnaire (AIS)**: The Illness Acceptance Scale was developed by Felton, Revensson, and Hinrichsen [33]. The scale can be used for any disease and measures the level of illness acceptance in adult patients. It indicates the individual’s acceptance of the disease through their reactions and feelings related to the illness and its treatment. The scale consists of eight statements describing negative consequences, including feelings of limitation due to the illness, personal inadequacy, dependency on others, and decreased self-esteem in accepting their illness. A low score indicates non-acceptance or poor adaptation to the illness and may be associated with negative emotions. Respondents’ answers range on a 5-point Likert scale, with scores from 1 to 5 as follows: 1- Strongly agree, 2- Agree, 3- Don’t know, 4- Disagree, and 5- Strongly disagree. The total score is the sum of scores across the statements, ranging from 8 to 40, indicating the level of illness acceptance. Scores below 20 are considered low and indicate non-acceptance or poor adaptation to the illness and associated emotional problems. Scores between 20 and 30 indicate a moderate level of illness acceptance. Scores above 30 indicate high or complete acceptance of the conditions by the individual. The reliability coefficient, Cronbach’s alpha, is 0.85.

**4- General Self-Efficacy Scale** (**GSE-17)**: This tool consists of 17 questions, which measure beliefs in one’s capability to handle new and difficult tasks [34] and each question is adjusted on a Likert scale ranging from “Strongly Disagree” to “Strongly Agree.” The scoring for each item ranges from 1 to 5. Questions 1, 3, 8, 9, 13, and 15 are scored from right to left, while the remaining questions are scored inversely, from left to right. Therefore, the maximum score a person can obtain from this scale is 85, and the minimum score is 17 [35]. The scale is unidimensional, with all items loading onto a single factor [34]. This scale has been translated and validated by Asgharnejad in Iran [36]. The reliability of this instrument was tested using Cronbach’s α and was found to be 0.86 [37].

### Statistical analysis

After collecting and encoding the data, they were entered into SPSS version 24 software. Initially, frequency, percentage, mean, and standard deviation indices were determined using descriptive statistics. The normal distribution of the data was assessed using the Kolmogorov-Smirnov test, skewness, and kurtosis. Although the data were slightly skewed, they were considered normal because the skewness and kurtosis were between − 1 and 1. ANOVA and T-test were used to determine the association between demographic and midwifery variables with the main research variable (quality of life). Pearson correlation was used to test the main hypotheses of the research (the existence of a relationship between self-efficacy and disease acceptance with quality of life). Additionally, linear single-variable and multiple-variable regression with the Enter method were used to examine predictive factors of quality of life. The significance level in this study was set at *P* < 0.05.

## Results

### Sample characteristics and their relationships with main variables

Participants consisted of 240 pregnant women diagnosed with diabetes during pregnancy. According to Table 1, the mean age of the participants was 32.13 years (SD = 5.98, range = 17–45). The majority of women had education levels below diploma (55.4%) and were homemakers (69.9%). The mean gestational age of the participants in the study was 27.60 weeks (SD = 8.09, range = 10–40), with more than 49.2% of them in the third trimester of pregnancy, and approximately two-thirds of pregnant women were multiparous. According to the findings, more than half of the cases were under dietary management for the treatment of gestational diabetes, and about half of them reported good health status. However, the majority of participants expressed their knowledge about diabetes as average to poor (Table 1).

Initial analysis of the association between demographic characteristics and the quality of life of diabetic pregnant women showed statistically significant differences in the mean quality of life score among different age groups, with women over 20 years of age having higher scores compared to women under 20 years (*P* = 0.020). Additionally, the mean quality of life score was higher in employed women (employees and self-employed) compared to homemakers (*P* < 0.001) (F = 14.643), women with university education compared to those with diploma and lower education (*P* < 0.001) (F = 12.118), and individuals who reported higher income levels compared to other groups (*P* < 0.001) (F = 12.204), and these differences were statistically significant. However, no statistically significant difference was found between gestational age groups and the number of pregnancies in terms of the mean quality of life score.

Regarding health status and knowledge about diabetes, the results showed that individuals who reported very good health status had higher quality of life scores compared to those who considered their health status as average and poor (*P* = 0.003) (F = 5.834), and pregnant women with high knowledge about diabetes also obtained higher quality of life scores compared to individuals with lower knowledge (*P* = 0.01) (F = 4.680), and these differences were also statistically significant. Finally, pregnant women under dietary management had statistically significantly higher mean quality of life scores compared to those who used insulin for treatment (58.55 vs. 55.8, *P* = 0.002).

**Table 1:** Participants’ demographic and disease characteristics and relationship with quality of life (*N* = 240)

Variable	*N* (%)	Mean (SD)	F/t	*P*-value
**women’s age groups (year)**			3.347	0.020
> 20	11 (4.6)	51.29 (5.67)		
20–29	52 (21.7)	57.43 (5.61)		
30–39	165 (68.8)	57.75 (6.77)		
40–49	12 (5.0)	57.21(7.62)		
**women’s educational status**			12.118	< 0.001
Primary & Secondary school	133 (55.4)	55.89 (6.29)		
Diploma	59 (24.6)	57.58 (6.29)		
University	48 (20.0)	61.14 (6.56)		
**women’s employment status**			14.643	< 0.001
Housewife	166(69.2)	56.00 (6.25)		
Employed	46(19.2)	61.59 (6.10)		
Self-Employed	28(11.7)	58.42 (6.81)		
**Gravid**			2.096	0.101
1	62 (25.8)	55.98 (6.27)		
2	83 (34.6)	58.09 (6.00)		
≥ 3	95 (39.6)	57.70 (7.25)		
**Gestatinal age**			0.287	0.751
> 14	15(6.3)	57.16 (5.43)		
15–28	107(44.6)	57.02 (7.03)		
> 28	118(49.2)	57.68 (6.44)		
**Income status**			12.204	< 0.001
More than enough	2 (0.8)	72.22 (3.92)		
enough	132 (55.0)	58.54 (6.67)		
Less than enough	106 (44.2)	55.52 (5.92)		
**Self-reported health**			5.834	0.003
Very good	2 (0.8)	72.22 (3.92)		
Good	128 (51.3)	57.69 (7.23)		
Moderate/Poor	115 (47.9)	56.74 (5.66)		
**Self-reported knowledge on diabetes**				
High	66 (27.5)	59.45 (6.88)	4.680	0.010
Moderate	93(38.8)	56.68 (6.63)		
Poor	81)33.80(	56.43 (6.12)		
**Diabetes treatment method**				
Diet	135 (56.3)	58.55 (6.00)	3.166	0.002
Diet and insulin	105 (43.8)	55.81 (7.1)		

Basic descriptive statistics regarding the main study variables, including quality of life, self-efficacy, and illness acceptance, are presented in Table 2. The mean score for quality of life was 57.36 (SD = 6.63, range 38.89-75), with the mean (standard deviation) of the physical domain of quality of life obtaining a lower score of 14.23 (2.30) compared to the psychological domain of 18.43 (2.17). Additionally, the descriptive statistics showed that the mean (standard deviation) scores for self-efficacy and illness acceptance were 51.75 (7.44) and 29.07 (7.69), respectively.

Furthermore, the results of Pearson correlation analysis between the main study variables can be observed in Table 2. According to the Pearson correlation analysis, it was found that the correlation between self-efficacy and overall quality of life score is statistically significant, positive, and of moderate magnitude (*r* = 0.457, *P* < 0.001). Moreover, the results indicated that the relationship between illness acceptance and quality of life in diabetic women is positively and significantly correlated (*r* = 0.385, *P* < 0.001), meaning that as self-efficacy and illness acceptance increase, the quality of life score of individuals also increases. Additionally, a positive and significant correlation was observed between self-efficacy and illness acceptance (*r* = 0.661, *P* < 0.001).

Finally, the correlation between self-efficacy and the psychological health domain of quality of life was found to be positive (0.291, *p* < 0.001), and with the physical health domain, a positive correlation (0.263, *p* < 0.001) was observed. The correlation between illness acceptance and the psychological health domain (0.302, *P* < 0.001) was inverse, while with physical health, it was direct (0.435, *P* < 0.001).

**Table 2:** Descriptive and correlations between the main variables of the study

Variable	Mean (SD)	Min-Max	1	2	3
1- Total SF-12 score	57.36 (6.63)	38.89-75.00	1	0.457*	0.385*
2-Total CD-RISC	51.75 (7.44)	31.00–73.00		1	0.661*
3-AIS	29.07 (7.69)	8.00–40.00			1

^*^P < 0.001

Table 3 presents the results of single-variable and multiple-variable regression analyses. According to Model 1, using single-variable regression analysis, there was a significant statistical relationship between self-efficacy and illness acceptance with quality of life. These variables alone could predict 20.6% and 14.4% of the variation in quality of life, respectively. Specifically, with each standard deviation increase in the score of self-efficacy and illness acceptance, the quality of life increases by 0.457 and 0.385 standard deviations, respectively.

Multiple-variable regression analysis in Model 2 indicated that 21.5% of the variation in quality of life could be explained by two variables, self-efficacy, and illness acceptance (0.21, *P* < 0.001 = R2adj), with only self-efficacy showing a significant relationship with quality of life and exerting the greatest influence on the quality of life variable (*P* < 0.001, β = 0.360).

Finally, in Model 3, by entering all variables into the regression model using the Enter method, the results showed that 23.3% of the variation in quality of life could be explained by the six entered variables (0.233, *P* < 0.001 = R2adj). Self-efficacy, illness acceptance, and income status had significant relationships with the dependent variable, with self-efficacy (*P* < 0.001, β = 0.280), illness acceptance (*P* = 0.033, β = 0.171), and income status (*P* = 0.028, β = 0.154) having the greatest impact on quality of life. Moreover, in Model 4 of the regression analysis, by including the two main variables (self-efficacy and illness acceptance), demographic characteristics, and three variables related to disease knowledge, personal health status, and type of treatment, the results indicated that all these variables could explain 25% of the variation in the quality of life of participating women, with high-income status allocating the most influence among the variables (Table 3).

**Table 3:** Univariate and multivariate linear regression analysis of quality of life

Predictors	Model summary					B	S.E	β	95%CI
	*R*	*R* ^2^	*R* ^2^_adj_	*P*-Value	F				
Model 1									
Total CD-RISC	0.457	0.209	0.206	< 0.001	62.887	0.407	0.051	0.457	0.306 to 0.509
AIS	0.385	0.148	0.144	< 0.001	41.364	0.332	0.052	0.385	0.230 to 0.434
**Model 2**	0.470	0.221	0.215	< 0.001	33.641				
Total CD-RISC				< 0.001		0.321	0.068	0.360	0.187 to 0.455
AIS				0.056		0.127	0.066	0.147	-0.003 to 0.256
**Model 3**	0.503	0.253	0.233	< 0.001	13.025				
Total CD-RISC				0.001		0.249	0.072	0.280	0.107 to 0.391
AIS				0.033		0.147	0.069	0.171	0.012 to 0.282
Women’s Age				0.252		0.073	0.064	0.066	-0.053 to 0.198
Women’s Educational Status				0.942		-0.020	0.274	-0.05	0.519 to 0.281
Women’s Employment Status				0.458		0.471	0.634	0.049	-0.777 to 1.719
Income Status				0.028		1.996	0.901	0.154	0.222 to 3.771
**Model 4**	0.528	0.279	0.250	< 0.001	9.796				
Total CD-RISC				< 0.001		0.281	0.074	0.316	0.136 to 0.426
AIS				0.233		0.110	0.092	0.128	-0.071 to 0.291
Women’s Age				0.097		0.007	0.071	0.006	-0.132 to 0.146
Women’s Educational Status				0.512		0.200	0.305	0.053	-0.401 to 0.801
Women’s Employment Status				0.399		0.545	0.646	0.057	-0.727 to 1.817
Income Status				< 0.001		4.583	1.282	0.354	2.056 to 7.110
Self-reported health				0.013		3.550	1.412	0.276	0.767 to 6.332
Self-reported knowledge on diabetes				0.683		0.309	0.758	0.036	-1.184 to 1.803
Diabetes treatment method				0.881		-0.172	1.147	-0.013	-2.432 to 2.088

Finally, the calculation of the effect of self-efficacy on illness acceptance in the single-variable regression indicated that the self-efficacy variable could predict 43.5% of the variation in the illness acceptance variable (0.435, *P* < 0.001 = R2adj), with a significant path coefficient of 0.661 (*P* < 0.001, β = 0.661). According to Table 4, the total effect of self-efficacy on the quality of life of pregnant women with diabetes was measured at 0.711.

**Table 4:** Standard coefficients of direct and indirect effect and total effect of self-efficacy on quality of life

Variable	effect		Total effect
	direct	indirect	
**Acceptance of illness**	0.385	-	-
**Self-efficacy**	0.457	0.254	0.711

## Discussion

The diagnosis of diabetes during pregnancy can significantly impact various aspects of a pregnant woman’s life and may bring along undesirable effects. Among the negative effects reported in previous studies are changes in mood, perceived health, and loss of control over oneself and life [9, 10, 38]. Given the increasing importance of quality of life, conducting multiple studies to examine influential factors is necessary. Research, especially in pregnant women facing diseases such as diabetes, is crucial for receiving appropriate interventions to improve maternal and neonatal outcomes [39, 40]. Diagnosing such diseases during pregnancy can greatly influence their lifestyle and have negative effects on various aspects of their lives, thus altering their perception of health and quality of life. However, self-efficacy and illness acceptance by pregnant mothers may potentially serve as buffers for the quality of life of pregnant women. This study aimed to examine the association between self-efficacy and the mediating effect of illness acceptance on the quality of life in pregnant women diagnosed with diabetes during pregnancy, and the main findings indicated a direct relationship between self-efficacy, illness acceptance, and quality of life in these women. Overall, the quality of life variable was explained by self-efficacy, illness acceptance, and income status.

To achieve the study objectives, the first step was to assess the quality of life of pregnant women with diabetes, which indicated an undesirable status of the participant’s quality of life in this study, with their overall quality of life score being average. The results obtained from other studies also indicate poor quality of life among women with high-risk pregnancies compared to women with normal pregnancies [41–43]. Additionally, researchers studying women and children with diabetes have reported low quality-of-life scores and highlighted the negative effects of diabetes on their quality of life. Dalfra et al. [44] reported that women with gestational diabetes mellitus (GDM) had lower quality of life scores compared to women with type 1 diabetes and women with normal pregnancies. Researchers have emphasized the negative effects of diabetes on health perception [45, 46]and have shown that it interferes with positive pregnancy experiences [47], thereby affecting quality of life due to its negative effects [10, 48].

The analysis of the relationship between demographic characteristics and quality of life among women with diabetes revealed a correlation with age, education, employment status, and income. Additionally, women with higher education levels achieved better quality of life scores than those with lower education levels. Women over 20 years old obtained higher quality of life scores compared to women under 20 years old. Researchers suggest that a potential explanation for the effect of age on quality of life is that younger patients may experience more stress regarding the future progression of the disease, which can negatively affect their mental health [49]. However, Iwanowicz-Palus et al. did not demonstrate statistically significant correlations between the age of the pregnant women studied and individual QoL [9]. Additionally, employed women scored higher than other groups (self-employed and homemakers), and having a very good income status positively impacted the quality of life score. This was also one of the predictor variables in the regression model of the current study.

Another finding was that individuals with a good health perception and high knowledge about diabetes also had higher overall quality of life scores compared to others. Bien et al. [10] also found similar results, reporting that women with excellent financial status, perceived good health, and average knowledge had higher quality of life scores. Additionally, those who managed solely through diet reported better quality of life compared to individuals treated with both diet and insulin. One of the obstacles to independence in dealing with a chronic disease is the patient’s financial status [10, 50, 51]. The authors’ assessment revealed that having a strong financial status can significantly impact various physical, psychological, and environmental aspects of an individual’s life, and financial resources are considered an important factor in a patient’s health. The diagnosis of diabetes usually entails increased costs of care, including medication, tests, and dietary restrictions, which can affect the family’s financial burden and the patient’s psychological status, as reported by other researchers, who stated that treatment costs increase the financial burden on the family [50, 52, 53] and can affect the quality of life [54]. However, contradictory results have been reported by Felicio et al., [49] who found that individuals with type 1 diabetes and poor economic status had the highest Health-Related Quality of Life. According to them, healthcare services can increase or enhance health inequalities. In this context, two variables need to be considered: access to and quality of health services. Also, the researcher reported pregnant women reporting very good living conditions had the highest scores for overall quality of life, general health, and quality of life in all specific WHOQOL-BREF domains [9]. Differences in studies in this area may stem from variations in the research population and cultural factors related to society. In the study by Felicio et al., [49] those with fewer complications from type 1 diabetes had higher quality of life scores, indicating that individuals with lower income may have fewer disease-related complications. The next issue is the reported high quality of life scores by women with type 2 diabetes who have college/university education and are employed. It appears that education and having optimal knowledge contribute to increased confidence, security, and better relationships with others. In our study, professionally active women reported a better quality of life. Similar results have been reported in other studies on patients with type 2 diabetes [52, 55] or Parkinson’s disease [55, 56]. Individuals with chronic diseases, including pregnant women with hyperglycemia, who remain professionally active, seem to have better access to information and medical care as well as a greater sense of physical and psychological security [9, 17, 44]. Higher scores were reported by pregnant women with hyperglycemia who had perceived good health statuses, which has also been reported in other studies [3, 10]. This could be due to the severity of the disease and the presence of disease-related complications. In our study, the type of treatment used was associated with perceived quality of life in pregnant women. The lowest quality-of-life scores were obtained by those treated with insulin. These findings have been reported by other researchers, who showed that insulin use significantly reduced the reported quality of life in pregnant women [14, 43]. The authors stated that the lower quality of life is only observed at the beginning of insulin treatment and that fear of injection and incorrect insulin administration can be effective factors, suggesting that sufficient education can increase the reported quality of life [47]. However, two other studies by Dalfra et al. [44] and Rodríguez-Almagro et al. [55] reported different results, showing that insulin treatment does not decrease the quality of life in pregnant and non-pregnant women with diabetes. According to the authors, better blood sugar control in individuals receiving insulin injections compared to diet therapy alone may be the reason [52, 57, 58].

In the present study, the relationship between two independent variables, self-efficacy and disease acceptance, with quality of life as the indicator showed a moderate correlation between these variables. With an increase in the level of self-efficacy and disease acceptance in patients, their quality of life also increased directly, in the regression model, the predictive ability for quality of life by these two variables was confirmed, with both being able to explain 21.5% of the variance in quality of life. Moreover, based on the regression model, three variables—self-efficacy, illness acceptance, and income status—accounted for 23.3% of the total variance in quality of life among pregnant women with gestational diabetes, with self-efficacy emerging as a predictive factor. What is recorded is that the psychological source influencing patient health behaviors is self-efficacy. This term refers to the belief in one’s ability to change behavior to cope with life’s challenges which is a key determinant of behaviors aimed at improving or maintaining individual health. Individuals with high levels of self-efficacy usually set higher goals for themselves and pursue them with greater diligence, having better self-monitoring and caregiving skills in conditions such as chronic illness [59, 60]. In the present study, the mean score on the self-efficacy questionnaire was 51.75, indicating a moderate level of self-efficacy in the women studied. Similar results have been reported in other studies in Iran for individuals with diabetes [61] and pregnant women [41]. Similar findings were also reported by Linden et al. (2016), [62] who examined self-efficacy in women with type 1 diabetes early in pregnancy. Bernal et al. (2000) reported that behaviors requiring problem-solving resulted in lower self-efficacy scores [63]. Self-monitoring blood sugar and performing disease-related care requires awareness, skills, and various resources. The patient needs to know when and how to self-monitor. The skill of measuring blood sugar levels and ultimately having the means to do so can be challenging, as providing all the necessities is somewhat difficult. Patients’ perceived self-efficacy may also decrease. Literature reviews have shown that other researchers have also found a relationship between self-efficacy and quality of life in individuals with diabetes [9]. However, in a study by Ghorbani et al. (2020) in Iran [64], a direct relationship between self-efficacy and quality of life was not observed. They indicated that self-efficacy can indirectly impact the quality of life by influencing self-care behaviors. Other studies have also shown that quality of life affects general self-efficacy in pregnant women with hyperglycemia, with those who rated their physical, psychological, social, and environmental quality of life higher also having higher self-efficacy scores. Additionally, according to reports by Linden et al. [62]and Weber-Rajek et al., [65] there is an association between high self-efficacy and better quality of life in women with type 1 diabetes early in pregnancy and among patients after ischemic stroke. Moreover, in this study, self-efficacy emerged as one of the most influential predictor variables.

Acceptance of the disease indicates the patient’s adaptation to their condition and anticipation of it. It is a factor that pertains to how an individual perceives and approaches their illness and the limitations it imposes [66], ultimately influencing their attitude towards treatment. Patients with a high level of disease acceptance adapt well to the conditions presented and cope with necessary lifestyle changes related to a disease, especially a chronic one [67, 68]. In the present study, the average score of disease acceptance, like two other variables, was moderate, consistent with the results of other studies [3, 69]. However, the literature review suggests that the comparison of average levels of disease acceptance is higher in pregnant women with gestational diabetes compared to other diabetic patients [68, 70]. This may be due to the special conditions of pregnancy that pregnant mothers experience to maintain their health and that of the fetus. As previously mentioned, the current study demonstrated a relationship between illness acceptance and quality of life in pregnant women. Women with gestational diabetes had higher scores on the overall quality of life associated with higher acceptance. This finding is similar to the results of other researchers such as Bien et al. [10] and lwanowicz‑Palus et al. [9]. A similar report was also published by Schmit and colleagues (2018) [71], which showed that low levels of disease acceptance lead to a decrease in the quality of life in diabetic patients. Finally, despite observing the relationship between self-efficacy and disease acceptance in the present study, the results indicate the indirect effects of self-efficacy on quality of life through the mediating variable of disease acceptance. Self-efficacy is a more important variable in coping with chronic disorders and plays a significant role in adapting to a wide range of symptoms of chronic diseases [72, 73]. It is reported to play a more important role than biomedical variables [73, 74], which was confirmed in the present study.

### Strengths and limitations of the study

The strength of our study lies in the fact that very few studies have been conducted on the quality of life and their determining factors in pregnant women with gestational diabetes, so we focused on the quality of life of pregnant women with gestational diabetes in light of their Self-efficacy as an individual’s belief and illness acceptance. According to this study, Future studies can be undertaken and other influential factors can be investigated, as the main study variables explain only a small portion of the quality of life. Additionally, a limitation of our study is its cross-sectional nature, which may lack the ability to express any causal relationships. Further research is needed to examine factors affecting the quality of life in pregnant women with gestational diabetes in a broader context.

## Conclusion

Pregnant women with gestational diabetes had a relatively moderate level of quality of life, self-efficacy, and disease acceptance. Sociodemographic factors such as maternal age, education level, employment status, and living conditions, self-reported knowledge of diabetes, and income status were reported to be associated with the quality of life of pregnant women, while self-efficacy and disease acceptance had a direct relationship with the quality of life score. Given the average scores in the main study variables and the explanation of only one-fourth of the status of the quality of life of pregnant women with gestational diabetes and gestational diabetes by self-efficacy, illness acceptance, and income status variables, it seems necessary for the maternity care team to take action and interventions needed to identify other factors affecting the quality of life, identify the individual needs of patients, and plan optimal interventions to optimize midwifery care for women with gestational diabetes during pregnancy and help them increase and improve their quality of life. Overall, care for women with gestational diabetes for improving their quality of life should encompass efforts to understand their expectations, promote health education, and address any challenges in self-care and self-monitoring. Proper management of these aspects by the treatment team can help optimize obstetric care for women with hyperglycemia, thereby improving their quality of life and level of illness acceptance and self-efficacy.

## Data Availability

The datasets are available from the corresponding authors on request”.

## Abbreviations

HRQoL: Health-related quality of life
AIS: Acceptance of illness scale
GSE-17: General self-efficacy scale − 17
T2DM: Diabetes mellitus type 2
